# Caveolin-1 is Associated with Tumor Progression and Confers a Multi-Modality Resistance Phenotype in Pancreatic Cancer

**DOI:** 10.1038/srep10867

**Published:** 2015-06-12

**Authors:** Moumita Chatterjee, Edgar Ben-Josef, Dafydd G. Thomas, Meredith A. Morgan, Mark M. Zalupski, Gazala Khan, Charles Andrew Robinson, Kent A. Griffith, Ching-Shih Chen, Thomas Ludwig, Tanios Bekaii-Saab, Arnab Chakravarti, Terence M. Williams

**Affiliations:** 1 The Ohio State University Medical Center, Arthur G. James Comprehensive Cancer Center and Richard J. Solove Research Institute, Columbus, OH 43210; 2 Hospital of the University of Pennsylvania, Philadelphia, PA, 19104; 3 University of Michigan Medical Center, Ann Arbor, MI, 48109; 4 Henry Ford Hospital System, West Bloomfield, MI, 48322

## Abstract

Caveolin-1 (Cav-1) is a 21 kDa protein enriched in caveolae, and has been implicated in oncogenic cell transformation, tumorigenesis, and metastasis. We explored roles for Cav-1 in pancreatic cancer (PC) prognostication, tumor progression, resistance to therapy, and whether targeted downregulation could lead to therapeutic sensitization. Cav-1 expression was assessed in cell lines, mouse models, and patient samples, and knocked down in order to compare changes in proliferation, invasion, migration, response to chemotherapy and radiation, and tumor growth. We found Cav-1 is overexpressed in human PC cell lines, mouse models, and human pancreatic tumors, and is associated with worse tumor grade and clinical outcomes. In PC cell lines, disruption/depletion of caveolae/Cav-1 reduces proliferation, colony formation, and invasion. Radiation and chemotherapy up-regulate Cav-1 expression, while Cav-1 depletion induces both chemosensitization and radiosensitization through altered apoptotic and DNA repair signaling. *In vivo*, Cav-1 depletion significantly attenuates tumor initiation and growth. Finally, Cav-1 depletion leads to altered JAK/STAT, JNK, and Src signaling in PC cells. Together, higher Cav-1 expression is correlated with worse outcomes, is essential for tumor growth and invasion (both *in vitro* and *in vivo)*, is responsible for promoting resistance to therapies, and may serve as a prognostic/predictive biomarker and target in PC.

Pancreatic carcinoma remains one of the most lethal cancers with a high mortality rate within the first year of diagnosis and a dismal 6% five year survival rate^1^. An estimated 46,420 new cases of pancreatic cancer will be diagnosed in 2014 in the U.S., of which approximately 39,590 people are estimated to die from this deadly disease^1^. Despite significant advancements in therapy, late presentation and rapid progression make it a difficult disease to treat and cure. The fibrotic nature of the stroma surrounding the tumor perhaps presents an appreciable barrier for therapies^2^,^3^, but the factors responsible for the aggressiveness of pancreatic cancer are still under investigation. This highlights the need for identification of useful biomarkers that would correlate pathological advancement of the disease and prognosis, and help select patients who would benefit most from multimodality treatment.

Caveolin-1 (Cav-1) is a 21 kDa protein found in “cave-like” invaginations of the cell membrane termed caveolae. Cav-1 is a major structural component of these 50-100 nm sized organelles, and is a requirement for their formation, as genetic ablation of Cav-1 renders many cell types devoid of caveolae^4^. Cav-1/caveolae are involved in various cellular pathways including, but not limited to, endocytosis, lipid homeostasis, and signal transduction^5^,^6^,^7^. Cav-1 has been implicated in the pathogenesis of oncogenic cell transformation and metastasis and has seemingly opposite roles of being either a tumor suppressor or an oncogene depending on the cancer type (breast or prostate) and tissue of interest (e.g. tumor or stroma)^8^,^9^,^10^,^11^.

In pancreatic cancer, Cav-1 is upregulated and has been correlated with poor prognosis or aggressiveness of the tumor^8^,^12^. Downregulation of caveolin-1 has been previously shown to sensitize cancer cells to ionizing radiation^13^. It has also been shown to mediate radioresistance in pancreatic cancer (PC) cells grown in three-dimensional culture^14^. Thus, Cav-1 expression in pancreatic cancer may be important for the prognosis of this disease.

In addition to surgical resection, current therapeutic strategies to treat pancreatic cancer include radiation and chemotherapeutics like gemcitabine, 5-fluorouracil, oxaliplatin, nab-paclitaxel, and irinotecan^15^,^16^. The identification of reliable biomarkers is needed to both prognosticate and potentially predict effective therapy for patients according to their tumor’s molecular profile. Here, we show that Cav-1 levels in pancreatic cancer could potentially serve as a prognostic biomarker. We establish roles for Cav-1 in both apoptotic and DNA damage response signaling in response to therapeutic stress. Finally, we demonstrate that targeting Cav-1 through genetic down-regulation in human pancreatic cancer cells can attenuate tumor growth and sensitize tumor cells to chemotherapeutics and radiation.

## Results

### Caveolin-1 is Overexpressed in Pancreatic Cancer ***In Vitro*** and ***In Vivo***, is Associated with Increasing Tumor Grade, CA19-9 Levels, and Worse Clinical Outcomes

**Figure 1:**
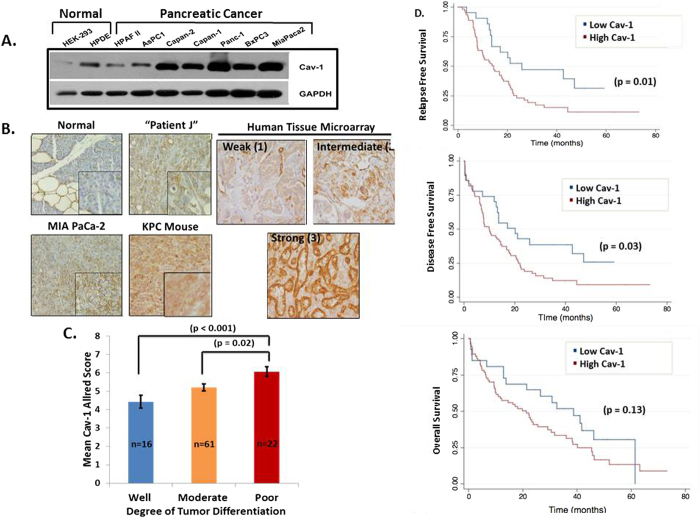
Cav-1 expression is up-regulated in pancreatic cancer and is associated with increased tumor grade and worse clinical outcomes. **A**. Western blot images showing expression of Cav-1 in normal and PC cells with GAPDH as a loading control. **B**. Left -Immunohistochemistry (IHC) staining of normal human pancreatic tissue (n = 4), xenograft tumor tissues derived from MIAPaCa-2 cells (n = 2), human patient-derived xenografts (n = 3), and tumors from KPC transgenic mice (n = 3), showing increased Cav-1 staining in pancreatic cancer compared to normal pancreatic tissue (inset at greater magnification). Right - Representative Cav-1 IHC staining of cores from a tissue microarray showing weak, intermediate, and strong intensity, with intensity scores of 1, 2, and 3 respectively. **C**. Mean Cav-1 IHC Allred score based on degree of tumor differentiation (well, moderate, poor). **D**. Low Cav-1 expression (Allred score 0-4) is significantly associated with improved RFS and DFS, with a trend towards improved OS, compared to high Cav-1 expression (Allred score 5-8).

Caveolin-1 has been implicated to have both pro-tumorigenic and tumor suppressive roles, depending on the tissue type and histopathology of tumors^5^,^17^. In pancreatic cancer, the role of Cav-1 is not well-defined. We determined Cav-1 expression in a panel of PC cell lines as well as normal human pancreatic ductal epithelium (HPDE) cells and HEK-293 kidney epithelial cells. Cav-1 expression was low in these normal cell lines, but highly up-regulated in 5 of 7 PC cell lines (Fig. 1A). Likewise, Cav-1 expression was virtually absent in normal human pancreatic epithelium, but expressed in neighboring adipose and endothelial cells, as expected (Fig. 1B). However, Cav-1 was clearly up-regulated in subcutaneous xenograft tumor tissue derived from MIAPaCa-2 cells, patient-derived xenografts, and tumors from a well-characterized mouse model of pancreatic cancer bearing p53 (p53^LSL-R270H^) and KRAS (KRAS^LSL-G12D^) mutations expressed in pancreatic epithelium with the *Pdx1-cre* transgene (“KPC mouse”) (Fig. 1B). Cav-1 expression was also tested on a tissue microarray of 110 patients with pancreatic cancer, and scored semi-quantitatively for low versus high expression. While Cav-1 is virtually absent in pancreatic ductal or acinar epithelial cells from which these tumors are derived (intensity score 0), 100% of 106 tumors with available staining data had some degree of Cav-1 staining: in the carcinoma cells, 60% were scored as weak (intensity score 1), 34% intermediate (intensity score 2), and 6% strong (intensity score 3) (Fig. 1B, right). Given the high preponderance of KRAS activating mutations in PC (~90%), we hypothesized whether KRAS mutations could be contributing to the high frequency of Cav-1 expression in pancreatic cancer. In order to address this, we utilized 2 independent isogenic cell line pairs (SW48 and DLD-1 cells), that were completely matched albeit due the presence of one mutated KRAS allele. Interestingly, the presence of a single mutated KRAS allele increased Cav-1 expression in these isogenic cell lines, perhaps accounting for the high frequency of Cav-1 up-regulation in PC (Supplemental Fig. S1). Taken together, while the majority of pancreatic cancers up-regulate Cav-1, the degree of this up-regulation varies between tumors.

Next, we correlated Cav-1 expression (dichotomized by low versus high scores) in the tissue microarray with clinical characteristics such as tumor histopathologic grade, serum CA19-9, and clinical outcomes including relapse-free survival (RFS), disease-free survival (DFS), and overall survival (OS). Higher Cav-1 expression was significantly associated with higher pre-operative CA 19-9 levels (a known poor prognostic marker in pancreatic cancer) by Pearson correlation statistical analysis (r = 0.235, p = 0.04; n = 74). In addition, higher tumor grade was significantly associated with increasing Cav-1 Allred score, indicating that Cav-1 is significantly up-regulated in more poorly differentiated tumors (Fig. 1C). Most importantly, higher Cav-1 expression is significantly correlated with worse clinical outcomes, such as worse relapse-free survival (p = 0.01), worse disease-free survival (p = 0.03), and a trend for worse overall survival (p = 0.13) (Fig. 1D).

### Cav-1 Downregulation Results in Decreased Proliferation, Invasion and Migration

**Figure 2:**
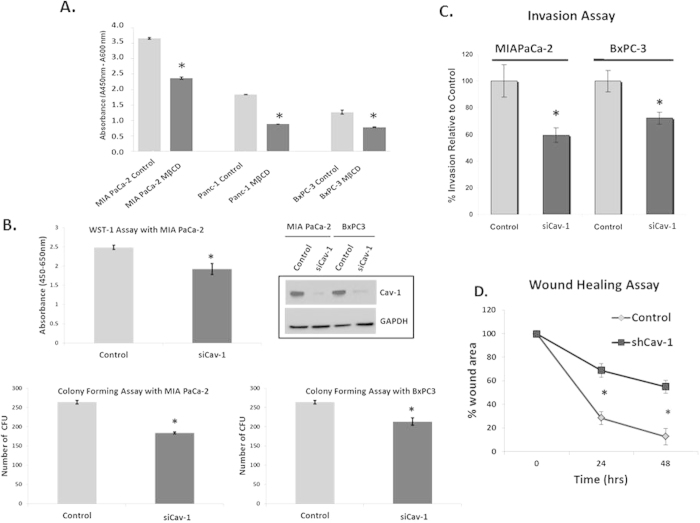
Cav-1 is essential for proliferation, invasion, and migration of pancreatic cancer cells. **A**. Pre-treatment of MIAPaCa-2, Panc-1 and BxPC3 cell lines with methyl-β-cyclodextran (MβCD) at 5 mM for 1 hour results in reductions in proliferation as measured by WST-1 assay (48 hrs). Absorbance is plotted on the y-axis where readings at 450 nm are subtracted from background reading at 600 nm, (*p < 0.05; n = 3). **B**. WST-1 assay comparing treatment with scrambled siRNA (“control”) and Cav-1 siRNA (“siCav-1”) in MIAPaCa-2 cells (top panel). Colony forming assays after treatment with scrambled control siRNA or Cav-1 siRNA on MIAPaCa-2 and BxPC3 cell lines (p < 0.05, n = 3, bottom panels). Western blot (top left) confirms appropriate Cav-1 depletion with Cav-1 siRNA in both cell lines. Virtually identical results were observed with a completely distinct set of pooled siRNA to Cav-1 (data not shown). **C**. Cells were seeded in 1% FBS-containing medium into the tops of Transwell chambers coated with Matrigel and incubated for 24 hours before being washed and fixed. Chemoattractant in the bottom well was 10% FBS. Columns represent percentage of invasion relative to control siRNA for different cell lines pre-treated with scrambled siRNA (control) and siCav-1 siRNA pools for 48 hours (left panel, p < 0.05, n = 4). **D**. Scratch (wound-healing) assay to measure migration, showing the percentage wound area plotted against time for scrambled siRNA (control)and siCav-1 treated MIA PaCa-2 cells (right panel). Note that control cells show almost complete closure of the “wound” compared to Cav-1 knockdown cells by 48 hours, suggesting a migration defect in Cav-1 depleted cells (*p < 0.05; n = 3).

Given the finding that Cav-1 is overexpressed in pancreatic cancer and is associated with worse outcomes, we determined whether or not caveolae could have a role in promoting pancreatic cancer tumor cell survival and proliferation. First, we disrupted caveolae with the cholesterol chelator methyl-beta-cyclodextran (MβCD), and performed WST-1 proliferation assays in multiple pancreatic cancer cell lines. Treatment with MβCD disrupted cell proliferation in all three cell lines, suggesting that caveolae have a role in PC cell proliferation (Fig. 2A). Since MβCD also disrupts other types of lipid rafts, we more directly assessed the role of Cav-1 in PC cell proliferation, by transfecting multiple PC cell lines with two distinct siRNA pools targeted against different conserved regions of Cav-1. Genetic downregulation of Cav-1 via siRNA resulted in reductions in cell proliferation and colony formation using multiple pooled Cav-1 siRNAs, compared with scrambled control siRNA, indicating that Cav-1 has specific roles in tumor cell proliferation (Fig. 2B). To further investigate this finding, cell cycle analysis was performed to determine whether loss of Cav-1 alters cell cycle distribution, but no substantial differences in cell cycle assortment were noted (Supplemental Table S1).

To evaluate the role for Cav-1 in PC cell invasion, we depleted Cav-1 from cells with siRNA or shRNA and performed Transwell invasion assays and wound healing (scratch) assays to evaluate the role of Cav-1 in these important tumor cell functions. Similar to previous reports, invasion and migration were decreased in response to Cav-1 down-regulation (Fig. 2C, D)^8^. These results indicate that Cav-1 supports pro-tumorigenic functions in PC cells including proliferation, invasion, and migration.

### Cav-1 is Up-Regulated After Genotoxic Chemotherapy or Ionizing Radiation, and Cav-1 Loss Sensitizes Cells to Genotoxic Therapy with Increased Activation of Apoptotic Response Pathways

**Figure 3:**
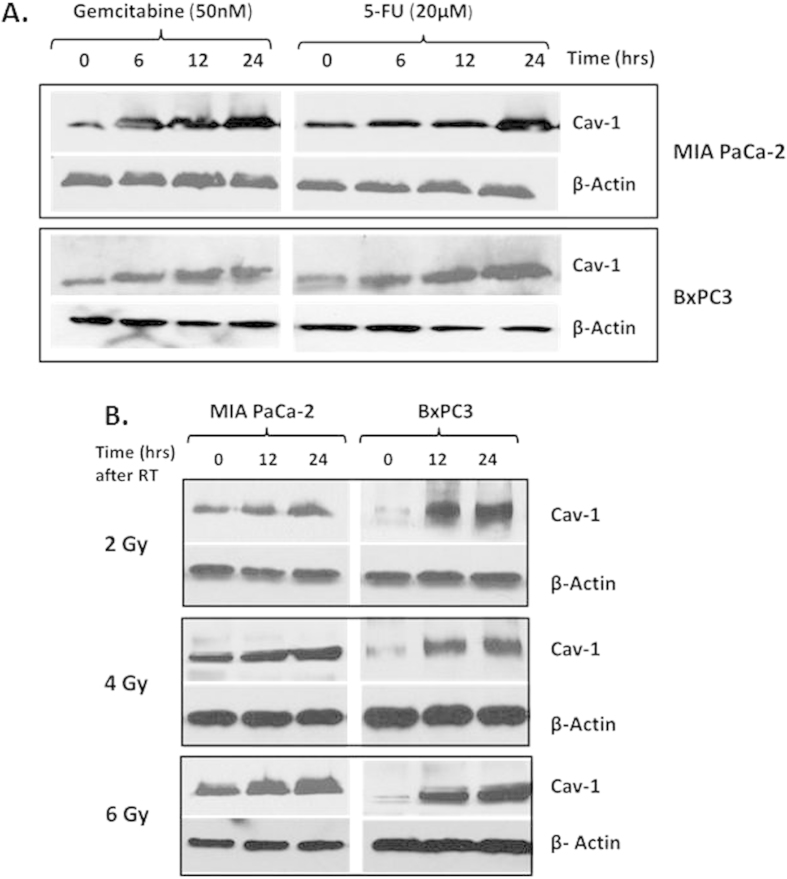
Cav-1 expression increases in response to chemotherapeutics and radiation. Immunoblotting for Cav-1 expression in MIAPaCa-2 and BxPC3 cell lines at various time points after treatment with **(A)** 50 nM gemcitabine or 20 μM 5-FU, or **(B)** after radiating with 2Gy, 4Gy or 6Gy. β-actin was used as a loading control.

In addition to surgical resection, two other therapies for PC include chemotherapy and radiation. Gemcitabine and 5-fluorouracil (5-FU) are two common chemotherapeutic agents widely used in PC with or without radiation. In order to assess a potential role for Cav-1 in therapy response, we first tested whether therapeutic stress with chemotherapy or ionizing radiation could alter levels of Cav-1 within PC cells. We found that commonly used chemotherapeutics used to treat pancreatic cancer increased Cav-1 expression (Fig. 3A). Similar to previous reports^13^,^14^, radiation also increases Cav-1 expression in a time-dependent manner in PC cells (Fig. 3B).

**Figure 4:**
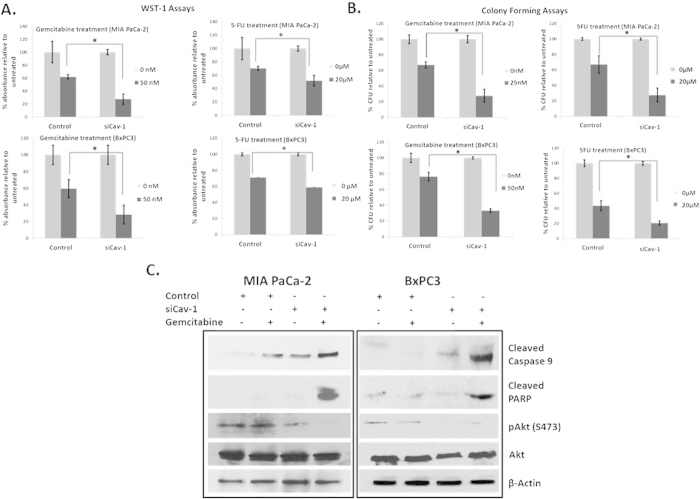
Cav-1 confers resistance to chemotherapeutics and Cav-1 knockdown leads to increased chemosensitivity. **A**. WST-1 assays showing cell proliferation with percentage absorbance of siCav-1 treated cells normalized to scrambled control siRNA with gemcitabine (50 nM) and 5-FU (20 μM) in MIAPaCa-2 cells (top panels) and BxPC3 cells (bottom panels). **B**. Colony forming assays with scrambled control siRNA or Cav-1 siRNA.Y-axis shows percentage of colony forming units (1 CFU = >50 cells) normalized to controls with gemcitabine (25 nM for MIAPaCa-2 and 50 nM for BxPC3) and 5-FU (20 μM) treatment on MIAPaCa-2 (top panels) and BxPC3 (bottom panels) cells. The lower concentration of gemcitabine was required for MIAPaCa-2 cells compared with WST-1 assay since too few colonies were noted at 50 nM. Treating with siCav-1 and chemotherapy resulted in decreased cell viability in both WST-1 and colony forming assays (*p < 0.05). **C.** Immunoblotting showing expression of cleaved caspase 9, cleaved PARP and pAkt in scrambled control siRNA and siCav-1 treated MIAPaCA-2 and BxPC3 cell lines treated with or without gemcitabine (50 nM).

Since Cav-1 is upregulated in PC cell lines after treatment with chemotherapeutics we hypothesized that Cav-1 could serve to protect PC cells from these therapies. Indeed, loss of Cav-1 sensitized multiple PC cell lines to both gemcitabine and 5-FU (Fig. 4A). These results were corroborated in colony forming assays (Fig. 4B). Treatment with even lower doses of 5-FU in the nanomolar range also resulted in attenuated proliferation in both cell lines where Cav-1 was transiently knocked down as compared to cells treated with scrambled control siRNA. (Supplementary Fig. S2). In order to determine whether loss of Cav-1 was affecting apoptotic signaling in response to chemotherapy, we investigated activation of apoptotic signaling pathways. After Cav-1 downregulation, treatment with gemcitabine led to increased levels of cleaved caspase-9, a key component of the intrinsic apoptotic pathway, and cleaved PARP (Fig. 4C). Akt activation was also reduced in response to Cav-1 depletion and was maximally reduced in response to the combination of Cav-1 siRNA and gemcitabine treatment, suggesting that Cav-1 may promote survival to chemotherapeutics in part through Akt activation. Together, these results suggest Cav-1 is important in promoting tumor resistance to chemotherapeutics in PC cells and that targeted Cav-1 knockdown sensitizes PC cells to genotoxic agents such as gemcitabine and 5-fluorouracil through activation of apoptosis and inactivation of pro-survival signals.

### Cav-1 Loss Sensitizes Cells to Ionizing Radiation, with Alterations in DNA Damage Response

**Figure 5:**
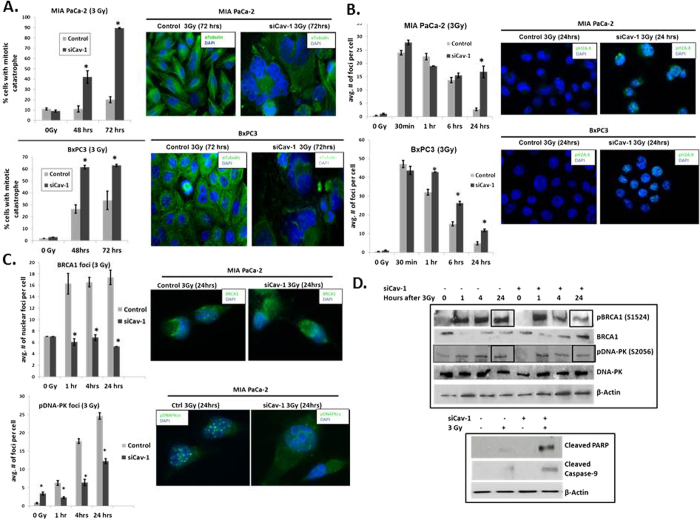
Cav-1 knockdown sensitizes tumor cells to radiation by increasing mitotic catastrophe and attenuating DNA damage response signaling **A**. Quantification of the percentage of cells with mitotic catastrophe after 3 Gy radiation (left panels, *p < 0.05). Representative immunofluorescence images of presence or absence of mitotic catastrophe at 72 hours in scrambled control and siCav-1 cells (right panels). **B**. Quantification of average number of nuclear pH2A.X foci per cell at multiple time points (*p < 0.05). Representative immunofluorescence staining for pH2A.X foci after 3 Gy radiation at 24 hours in scrambled control siRNA and siCav-1 cells (right panels). **C**. Top - Quantification of average number of nuclear BRCA1 foci per cell at multiple time points (left; *p < 0.05). Representative immunofluorescence staining of BRCA1 foci after radiation at 24 hrs in scrambled control siRNA vs siCav-1 (right panels). Bottom - Quantification of average number of nuclear pDNA-PK foci per cell (left; *p < 0.05). Representative immunofluorescence staining of pDNA-PK foci after radiation at 24 hrs in scrambled control siRNA-and siCav-1 (right panels). **D**. Immunoblotting of scrambled control and siCav-1 treated cells with or without radiation at different time points for expression of phosphorylated BRCA1, total BRCA1, phosphorylated DNAPK, total DNAPK, and beta actin as control (top panel). Note the reductions in phospho-BRCA1 and phospho-DNA-PK at 24 hrs after radiation with Cav-1 depletion. Loss of Cav-1 also increases the degree of radiation-induced activation of apoptotic signaling at 24 hours after 3Gy (bottom panel). For nuclear foci and mitotic catastrophe experiments, at least 50 cells were counted per datapoint.

As shown in Fig. 3B, Cav-1 levels were markedly upregulated in a time-dependent fashion, suggesting a role for Cav-1 in radiation response. Similar to previous published results^13^, we also found that Cav-1 down-regulation with siRNA sensitizes PC cells to radiation (Supplemental Fig. S3). Furthermore, Cav-1 depletion significantly increased the fraction of cells undergoing mitotic catastrophe after radiation as indicated by the presence of abnormal multi-lobulated nuclei, suggesting a role for Cav-1 in DNA damage response and/or repair (Fig. 5A). Likewise, Cav-1 down-regulation also resulted in delayed resolution of gamma H2A.X nuclear foci (phospho-H2A.X Ser139), most pronounced at 24 hrs after radiation (Fig. 5B). Together, these two findings suggest that Cav-1 loss results in deficiencies in DNA damage response or repair. In order to further investigate this, we assessed two major mechanisms of DNA double strand break repair after radiation: homologous recombination (HR) and non-homologous end joining (NHEJ)^18^,^19^,^20^,^21^. Given that BRCA1 is an important intermediary in HR repair^22^, we assessed BRCA1 focus formation. We observed a marked decrease in BRCA1 nuclear foci in Cav-1 knockdown cells after radiation (Fig. 5C, top). Since DNA-PK is an important component of NHEJ repair, we also assessed phosphorylated DNA-PK (S2056) focus formation after radiation. Similar to BRCA1, Cav-1 depletion resulted in a significant reduction in phosphorylated DNA-PK nuclear foci after radiation through most timepoints (Fig. 5C, bottom). In corroboration of these findings, Cav-1 loss results in decreased phosphorylated BRCA1 and DNA-PK by immunoblotting, particularly at 24 hrs after radiation (Fig. 5D, top). While defects in both HR and NHEJ signaling repair were noted after Cav-1 down-regulation, no significant changes in early activation of DNA damage response were found as measured by phospho-ATM S1981 (data not shown). Furthermore, Cav-1 siRNA induced higher levels of apoptosis pathway activation 24 hours after radiation (Fig. 5D, bottom). As mentioned previously, we did not detect any differences in cell cycle distribution with Cav-1 loss with or without radiation (Supplemental Table S1), indicating that changes in cell cycle distribution could not account for the differences observed: as expected, radiation induced a G2/M arrest, but loss of Cav-1 did not impart significant changes in the cell cycle assortment.

### Knockdown of Cav-1 Attenuates Tumor Growth in Mice

**Figure 6:**
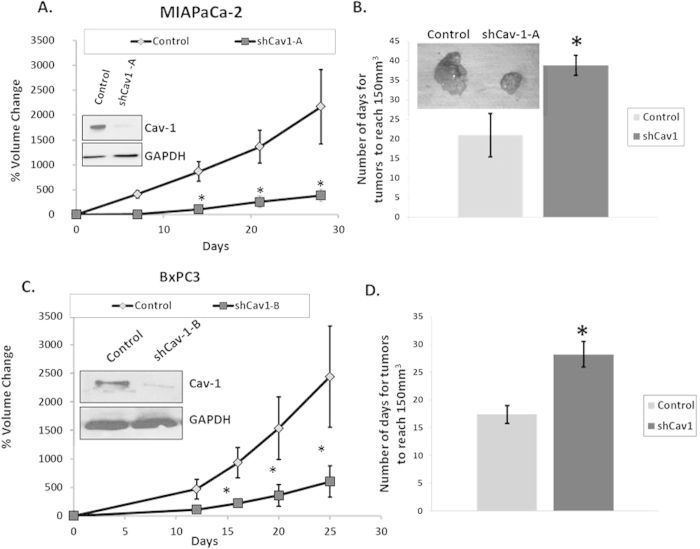
Cav-1 is essential for tumor growth and proliferation *in vivo*. Tumor growth curves for **(A)** MIAPaCa-2 shCav-1-A bearing and **(C)** BxPC3 shCav-1-B bearing xenografts in athymic nude mice measured by tumor volumes up to 30 days after injection of cells. Growth curves for xenografts resulting from scrambled control shRNA (“control”) cells for each cell line are also shown for comparison. Bar graph shows mean number of days for tumors to reach 150 mm^3^ for MIAPaCa-2-shCav-1-A **(B)** and BxPC3-shCav-1-B **(D)** xenografts. Insets in (A) and (C) are immunoblotting confirming Cav-1 down-regulation in the stable cell lines. Inset in (B) is a representative image demonstrating the difference in size of scrambled control shRNA versus MIAPaCa-2-shCav-1–A tumors at about 30 days after injection. For each cell line, at least n = 5 mice per study group were used; *p < 0.05.

Given our *in vitro* and human clinical outcomes data, we tested the role for Cav-1 in PC tumor initiation and proliferation *in vivo*, by injecting two different PC cell lines (MIAPaCa-2 and BxPC3) with stable Cav-1 knockdown using two independent shRNA constructs targeting Cav-1 (shCav-1-A in MIAPaCa-2, and shCav-1-B in BxPC3). Tumor initiation was markedly decreased in Cav-1 knockdown cells, with only 8 of 30 (27%) injected mice developing tumors, compared to 18 of 30 (60%) injected mice with control cells (p < 0.05, Fisher’s exact test). Tumor growth rates were also severely attenuated in mice bearing MIAPaCa-2 shCav-1-A xenografts compared to control (non-specific) shRNA xenografts (Fig. 6A,B). In order to determine whether differences in tumor initiation were related to the athymic model or incomplete immune suppression, we also injected NOD SCID and CB17 mice with control and shCav-1 MIAPaCa2 cells (n = 5 tumors per cell line). In confirmation of the athymic data, Cav-1 depletion resulted in 0/5 (0%) and 1/5 (20%) NOD-SCID and CB17 mice developing tumors, respectively, compared to 4/5 (80%) and 5/5 (100%) control mice (p < 0.05, Fisher’s exact test). With BxPC3 xenografts harboring a completely distinct shRNA (shCav-1-B) compared to the MIAPaCa-2 xenografts, there was likewise a substantial attenuation in growth rate (Fig. 6C,D). Due to the substantial effects of Cav-1 loss on tumor initiation and growth rate, we were unable to independently assess the effects of Cav-1 levels on chemotherapy or radiation response using these cell line models. Taken together, these results indicate that Cav-1 is essential for growth and progression of PC cells *in vivo* as well as *in vitro*.

### Knockdown of Caveolin-1 inhibits STAT3, Src, and JNK signaling in pancreatic tumor cells

**Figure 7:**
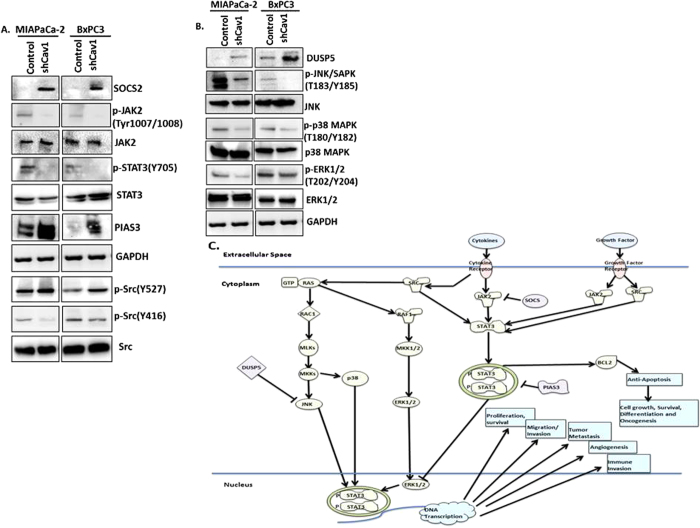
Knockdown of Caveolin-1 leads to decreased STAT3, Src and proliferative signaling *in vitro*. **(A)** shCav-1 downregulated cells in both MIAPaCa-2 and BxPC3 cell lines showed decreased phosphoJAK2 at residues Tyr1007/1008 and phosphoSTAT3 at Tyr705 as compared to scrambled control shRNA cells. Total levels of JAK2 and STAT3 are unchanged. SOCS2 and PIAS3, both inhibitors of the JAK-STAT pathway, are upregulated in Cav-1 knockdown cells. Phosphorylation of Src at Tyr416, the activation site, is decreased while phosphorylation of Src at Tyr 527, the inhibition site, is increased. GAPDH equal loading control remains unchanged. **(B)** Phosphorylation of JNK at Thr183/Tyr185, p38MAPK at Thr180/Tyr182 and pERK1/2 at Thr202/Tyr204 is decreased in both cell lines with knockdown of Cav-1, consistent with global reduction in proliferative pathways. Totals remain unaffected. DUSP5 levels are increased in both cell lines concomitantly. GAPDH shows equal loading control. **(C)** Schematic depicting the pathways affected in signaling when Cav-1 is downregulated in pancreatic cancer cell lines.

In order to better define a mechanism for how Cav-1 loss attenuates tumor cell growth, we performed comprehensive immunoblotting of various signaling pathways in our isogenically matched MIA-PaCa2 and BxPC3 cells. In doing so, we identified alterations in the JAK-STAT signaling pathway. Specifically, loss of Cav-1 leads to increased levels of SOCS2 (suppressor of cytokine signaling), a negative regulator of JAK-STAT signaling (Fig. 7A). Concordant with our finding, Cav-1 depletion leads to reduced activation of JAK2 and downstream STAT3 signaling (Fig. 7A). Furthermore, loss of Cav-1 was associated with increased PIAS3 (protein inhibitor of activated STAT3) levels, a protein which negatively regulates STAT3. Since the JAK-STAT pathway is an important regulator of cell survival and cytokine receptor signaling, our data suggests that knocking down caveolin-1 affects cell survival and proliferation in pancreatic cancer cells via this pathway. In addition, we identified that loss of Cav-1 led to decreased activation of the JNK/SAPK pathway, as well as p38 and p42/44 MAPK signaling, suggesting probable suppression of survival pathways. (Fig.7B).This may also explain the proliferation defect that we found initially when Cav-1 was knocked down (Fig. 2B). Concomitant with this finding, we found that Cav-1 depletion increased DUSP5 levels, a nuclear protein which negatively regulates JNK as well as p38 and ERK1/2^23^,^24^,^25^. Lastly, since Cav-1 was first identified as a substrate of v-Src, we queried whether loss of Cav-1 altered endogenous c-Src activation. We found that Cav-1 knockdown resulted in attenuation of Src signaling as observed by decreased Y416 phosphorylated Src (activated form), and increased Y527 phosphorylated Src (inhibited form) (Fig. 7A). Since JAK2, SOCS, JNK/SAPK, and Src signaling all converge and impinge on STAT3 signaling, our data suggests that loss of Cav-1 serves to reduce pancreatic cancer oncogenic proliferation, migration, invasion, and cell survival through a STAT3 mechanism (Fig. 7C).

## Discussion

Here, we have performed a comprehensive study elucidating diverse roles for Cav-1 in PC. Using human samples, we demonstrate that Cav-1 is a prognostic biomarker in pancreatic cancer. Our results also indicate that Cav-1 is essential for growth, invasion, and proliferation of PC cells both *in vitro* and *in vivo*. We also demonstrate that Cav-1 potentiates resistance to radiation and chemotherapeutics such as gemcitabine and 5-FU, suggesting that targeting this pathway may lead to improved therapeutic response. Finally, we establish a novel role for Cav-1 in a SOCS-JAK-STAT-SRC signaling pathway in pancreatic cancer cells.

Previously, Cav-1 expression has been correlated with increased tumor diameter, poor histopathologic grade, and poor prognosis in a clinical cohort of 79 Japanese PC patients. Interestingly, this study also demonstrated that expression of Cav-1 was not elevated in chronic pancreatitis, thus providing more data for Cav-1 as a suitable potential biomarker for invasive pancreatic carcinoma^26^. Another clinical study of fewer patients also found fatty-acid synthase (FAS) and Cav-1 are co-expressed in PC and are associated with poor survival, providing further evidence for Cav-1 as a potential PC biomarker^27^. Our data from a significantly larger clinical dataset similarly demonstrates Cav-1 to be associated with worse tumor histologic grade and clinical outcomes.

Prior reports have linked Cav-1 overexpression with oncogenic transformation, invasion, and metastasis^8^,^28^,^29^,^30^. We similarly found that Cav-1 knockdown by multiple distinct siRNAs reduced proliferative, invasive and migratory properties of PC cells in multiple cell lines. Disruption of lipid rafts by MβCD also led to a decrease in proliferation, suggesting intact lipid rafts and caveolae are essential for Cav-1 signaling. This change in rate of proliferation was not due to changes in the cell cycle as shown in Supplemental Table S1. Most importantly, our *in vitro* results were corroborated *in vivo*, as Cav-1 down-regulation markedly attenuated tumor initiation and growth rates in mice, providing direct evidence that Cav-1 is essential for tumorigenicity, growth and proliferation in pancreatic cancer. To our knowledge, the findings of such direct effects of Cav-1 on tumor initiation, growth and proliferation in pancreatic cancer cells is a novel finding.

Treatment by gemcitabine and 5-fluorouracil (5-FU), are two common chemotherapeutic approaches for PC. We found that increasing length of chemotherapy exposure increased Cav-1 expression perhaps in a “stress-like” response, and that Cav-1 depletion led to increased sensitivity to both chemotherapeutics, particularly gemcitabine. Activation of the intrinsic pathway of apoptosis is a common mode of tumor cell death under conditions of chemotherapeutic stress and involves activation of caspases like cleaved caspase 9^31^,^32^,^33^. Our results also indicate that Cav-1 knockdown sensitizes PC cells to chemotherapy through the intrinsic pathway of apoptotic cell death, with concomitant loss of Akt pro-survival signals. To our knowledge, a role for Cav-1 in promoting chemoresistance through protection from activation of the intrinsic pathway of apoptosis is a novel finding.

Similar to chemotherapeutics, radiation is commonly used in PC^34^,^35^,^36^,^37^. We have found that Cav-1 expression increased with time in multiple PC cell lines after radiation, similar to a previous report^13^. After radiation, phosphorylated H2A.X is recruited to sites of double strand DNA breaks, and itself recruits proteins to effect DNA repair^38^,^39^. We have extended our results to show that Cav-1 down-regulation leads to delayed repair of radiation-induced DNA damage, as demonstrated by delayed pH2A.X foci resolution. Delayed resolution of these foci suggests inhibition of repair, leading to accumulation of damaged DNA and an inability to properly divide following replication^40^,^41^,^42^,^43^. In confirmation of this, we observed an increased number of multi-nucleated cells indicating increased mitotic catastrophe in Cav-1 depleted cells after radiation, with subsequent clonogenic radiosensitization. Mechanistically, we observed that knocking down Cav-1 affected both HR and NHEJ processes of double strand break repair, similar to a previous report^21^. Radiation-induced Cav-1 has also been shown to be involved in nuclear translocation of EGFR with subsequent activation of DNA-PK, which may provide one possible mechanism for a role for Cav-1 in double strand break repair^44^. We have also provided novel data implicating Cav-1 in facilitating BRCA1 and DNA-PK mediated DNA repair and protection from intrinsic apoptosis after ionizing radiation.

Finally, our data suggests that Cav-1 depletion in pancreatic cancer cells affects various pathways, particularly the JAK/STAT pathway, by decreasing activation of JAK2 and STAT3 and by increasing SOCS2, PIAS3, and DUSP5 inhibitory signals. JNK has been another proliferation pathway involved in stress-induced proliferation and survival, but which has also been shown to promote STAT3 activation^45^. We find that loss of Cav-1 reduces JNK activation, perhaps through increased function of the JNK inhibitor DUSP5. In addition, Src and Src family tyrosine kinases are well-characterized caveolae-associated proteins, which have been shown to bind Cav-1,phosphorylate Cav-1, and be regulated by Cav-1^28^,^46^,^47^. Our data indicates that Cav-1 depletion results in reduced Src activity, providing further evidence that Cav-1 has pro-tumorigenic functions, perhaps through a direct association between Cav-1/caveolae and Src. Furthermore, Src has been shown to promote STAT3 activation and JNK^48^,^49^,^50^. Also, STAT3 has been shown to bind to the promoter region of Cav-1 in breast cancer^51^ and may thus result in a positive feedback loop if the same phenomenon occurs in pancreatic carcinoma. Thus, many signals in the Cav-1 signaling pathway appear to be converging on the STAT3 pathway. STAT3 is a well-characterized positive regulator of various cell survival and signaling pathways, through activation of transcriptional programs leading to tumor cell proliferation, survival, migration/invasion, metastasis, immune evasion, and tumor angiogenesis^52^. Overall, our data establishes a novel interaction between Cav-1 and both JAK/STAT3 and JNK signaling in tumor cells, and we postulate that Cav-1 and caveolae directly promotes activation of STAT3 and JNK pathway activation through Src. Future efforts will be directed at further clarification of this pathway.

In summary, our data demonstrate a pro-tumorigenic role for Cav-1 in PC cell proliferation, tumor initiation, tumor growth, invasion, migration and multimodality therapeutic resistance. Our studies also demonstrate a novel role for Cav-1 in promoting JAK/STAT and JNK activation in tumor cells. Further studies are required to better elucidate the signaling pathways downstream of Cav-1 involved in these processes and identify novel ways to directly or indirectly target the Cav-1/caveolae pathway in PC for therapeutic efficacy. Finally, prospective evaluation of Cav-1 as a prognostic and predictive biomarker in PC is warranted.

## Materials and Methods

### Antibodies, Chemicals, and Cell Culture

Anti-caveolin-1 (N-20) and DUSP5 antibody were purchased from Santa Cruz Biotechnology (Santa Cruz, CA). Anti- cleaved caspase-9, cleaved PARP, phospho-Akt, total Akt, phospho-BRCA1, phospho-DNAPK, total BRCA1, total DNAPK, phospho-JAK2, total JAK2, phospho-STAT3, total STAT3, PIAS3, Src (Y416), Src (Y527), total Src, SOCS2, phospho-JNK, total JNK, phospho-p38, total p38, phospho-ERK1/2, total ERK1/2, alpha tubulin, phospho-H2.AX, beta actin and GAPDH antibodies were purchased from Cell Signaling Technology (Danvers, MA). Gemcitabine and 5-fluorouracil (5-FU) was purchased from Sigma-Aldrich (St. Louis, MO) and Santa Cruz Biotechnology (Dallas, TX) respectively. For *in vitro* studies, gemcitabine and 5-FU were dissolved in DMSO. MIAPaCa-2, BxPC3, Panc-1, AsPC1, Capan-1, Capan-2, HPAFII and HEK-293 were obtained from and authenticated (via short tandem repeat profiling) by the American Type Culture Collection (Manassas, VA), and grown according to ATCC recommendations. HPDE cells were kindly provided by Dr Diane Simeone (University of Michigan). Cells used for this study were cryopreserved within 6 months of authentication. SW-48 and DLD-1 isogenic cell line pairs were obtained from Horizon Discovery (Cambridge, UK). Cells were passaged for no longer than 3 months and grown in a 37 °C incubator with 5% CO_2_.

### Caveolin-1 knockdown

Scrambled nonspecific control and anti-Cav-1 SMARTpool siRNA were purchased from Dharmacon (Lafayette, CO). A second set of siRNA targeting Cav-1 was purchased from SantaCruz Biotech (Dallas, TX). Transfections were performed according to manufacturer’s protocol using Oligofectamine (Invitrogen). Transient transfections were carried out 48 hours before drug treatment or radiation. For stable Cav-1 knockdown, cells were transduced with shRNA lentiviral particles (SantaCruz Biotech) and stable pools were selected with puromycin.

### Immunoblotting

Immunoblotting was performed as described before^53^. Briefly, cell lysates were prepared in RIPA lysis buffer (1% NP-40, 150 mM NaCl, 50 mM Tris-HCL pH 7.4, 0.25% Na-deoxycholate, 1 mM EDTA) supplemented with 1x protease inhibitor (Complete, Roche Applied Science) and phosphatase inhibitors (PhosSTOP, Roche Applied Science). Protein concentration was determined with a *Dc* Protein Assay Kit (BioRad, Hercules, CA). Proteins were resolved by SDS/PAGE and transferred to nitrocellulose membranes. Primary antibodies were allowed to bind overnight at 4℃, and used at a dilution of 1:500-1,000. After washing in TBS-Tween, membranes were incubated with horseradish peroxidase-conjugated secondary antibodies diluted 1:2,500 for 1 hour. Membranes were washed with TBS-Tween and incubated for 1 minute with enhanced chemiluminescence reagent (Amersham Pharmacia, Uppsala, Sweden) prior to film exposure.

### Cell Proliferation Assays

Cells were plated in 96 well format and treated with DMSO, gemcitabine or 5-FU for 48 hours before the assay. Cell proliferation assay was performed with WST-1 reagent (Roche) according to their protocol. Briefly, 10 μl of WST-1 reagent was added to each well after treatment and incubated at 37 °C for four hours. Absorbance was read at two wavelengths, 450 nm and 600 nm for endpoint absorbance and background absorbance, respectively. The absorbance difference was plotted on the y-axis with the treatment conditions on x-axis.

### Colony Forming Assays

Cells were trypsinized to generate single cell suspensions and seeded into 60 mm tissue culture plates in triplicate. Cells were incubated with DMSO or gemcitabine or 5-FU for a total of 24 hours before changing the medium. If irradiated, cells had medium changed 24 hours after radiation. At 7-14 days after seeding, colonies were stained with 0.5% crystal violet, and the numbers of colonies or colony forming units (CFU) containing at least 50 cells were counted. Experiments were repeated multiple, independent times.

### Experimental Radiation

Irradiation was performed essentially as described previously with 160 kV, 25 mA at a dose rate of approximately 113cGy/min using a RS-2000 biological irradiator (RadSource,GA)^53^.

### Immunofluorescence

Cells were plated on coverslips and treated with chemotherapeutics or radiation, then fixed with 2% paraformaldehyde for 15 minutes at room temperature, rinsed with PBS and permeabilized with 1% Triton-x-100 for 10 min on ice. After rinsing, cells were blocked with 3% bovine serum albumin for 1 hour or overnight at 4 °C. In a humidified chamber, primary antibodies were added in blocking solution and incubated for various times, rinsed, and secondary antibody was added along with DAPI for 1 hour at room temperature. Cells were then rinsed, mounted with coverslips, and sealed until visualization with a confocal microscope.

### Flow cytometry

Flow cytometry was performed as described previously^53^. Briefly, cells were plated in 6 well dishes, and treated with or without radiation. At 24 hours following radiation exposure, cells were harvested with trypsin, washed with medium, centrifuged, and resuspended in 1 mL PBS. Cells were immediately fixed for 30 minutes with 9 ml ice-cold 80% ethanol added slowly with mixing. Cells were washed twice with PBS and resuspended in 1 mL PBS containing 10 μg/mL propidium iodide and 0.25 mg/mL RNase A. Cells were incubated for 30 minutes and stored in the dark prior to analysis. Cells were then transferred to 5 mL polystyrene round bottom tubes through cell strainer lids and analyzed on a FACS Calibur (BD Biosciences). Data was fit using ModFit *LT* (Verity Software House).

### Immunohistochemistry

Immunohistochemistry was performed as described before^53^. Briefly, slides were deparaffinized in xylene (3 changes of 2 minutes each) and then rehydrated through graduated alcohols of 2 minutes each (100%, 95%, and 70%) followed by distilled water. The slides were placed in a 3% peroxidase block for 5 minutes, followed by antigen retrieval for 25 minutes in pH 6.0 citrate buffer in a microwave, cooled and washed in distilled water. After rinsing in TBS, slides were incubated with a primary antibody for caveolin-1 (N-20, SantaCruz) at 1:200 for 60 minutes on a Biocare Medical Intellipath Autostainer. After primary antibody incubation, the slides were rinsed and incubated with a horseradish perodixase (HRP) Polymer two-step system with a conjugated rabbit secondary antibody for 20 minutes (Dako). Slides were rinsed again in TBS, incubated with DAB + (Dako) for 5 minutes, then rinsed and counterstained with hematoxylin for 10 seconds. Slides were then rinsed in ammonia water and dehydrated in graduate alcohols following the opposite order (70%, 95% and 100% alcohol) followed by xylene, and mounting with a coverslip.

### Animal experiments

*In vivo* experiments were conducted as described previously^53^. Six to eight week-old male nude mice (Taconic Farms Inc., NY) were caged in groups of five or less, and fed a diet of animal chow and water ad libitum. MIAPaCa-2 and BxPC3 stable cell lines bearing control or Cav-1 shRNA (2–4 × 10^6^ cells each) were injected subcutaneously into the flanks of each mouse. To obtain a tumor growth curve, perpendicular diameter measurements of each tumor were measured every 3 days with digital calipers, and volumes were calculated using the formula (L × W × W)/2. Animal studies were conducted in accordance with an approved protocol adhering to the IACUC policies and procedures at The Ohio State University.

### Tissue Microarray

A tissue microarray was created from 110 patients treated with surgery and chemoradiation for pancreatic cancer at the University of Michigan. All tissue cores were obtained from diagnostic or surgical samples, and the most representative, non-necrotic areas were chosen by an experienced pathologist (D.T.). All hematoxylin and eosin stained slides of the tumors were reviewed, and tissue microarrays were constructed as described previously^54^. Tissue cores (1.0 mm in diameter) were taken from spatially separate areas in a single donor block from each case using a tissue microarrayer (Chemicon Advanced Tissue Arrayer, Temecula, CA). Cores were arrayed into a recipient block at predetermined coordinates (2 cores per patient). The H&E stained sections from donor and recipient paraffin blocks were used to confirm the area of tumor from which cores were retrieved. After immunohistochemical staining, expression of Cav-1 was scored using the Allred method by an independent pathologist blinded to patient data (D.T.), by determining the average intensity score (0 = none, 1 = weak, 2 = intermediate, 3 = strong) and percent cells staining score (0 = none, 1 = <1%, 2 = 1–10%, 3 = 10–33%, 4 = 33–66%, 5 = >66%), and summing the scores for a range of 0-8^55^. Scores from each of the 2 cores were averaged. High and low Cav-1 expression was classified by dichotomizing Allred score from 0-4 (low) to 5-8 (high).

### Data Analysis

Data are presented as the mean ± standard error of the mean (s.e.m.) for clonogenic survival and tumor growth experiments. The group comparisons of the percent change in tumor volume were performed at individual time points. Statistical comparisons were made between the control and experimental conditions using the unpaired two-tailed Student’s *t*-test with significance assessed at *p*-values <0.05. Overall survival (OS), relapse-free survival (RFS), disease-free survival (DFS) were determined using the Kaplan-Meier method. RFS indicates percent of patients free of first relapse (whether local, regional, or distant). DFS indicates percent of patients free of any relapse or death. The log-rank test was used to determine if Cav-1 expression was associated with outcomes, with p < 0.05 considered significant. Patients lost to follow-up were censored. Stata, version 11 (StataCorp, College Station, TX) was used to perform the statistical analyses.

## Additional Information

**How to cite this article**: Chatterjee, M. *et al*. Caveolin-1 is Associated with Tumor Progression and Confers a Multi-Modality Resistance Phenotype in Pancreatic Cancer. *Sci. Rep*. **5**, 10867; doi: 10.1038/srep10867 (2015).

## Supplementary Material

Supplementary Information
