# Supplementary material for: Caveolin-1 is Associated with Tumor Progression and Confers a Multi-Modality Resistance Phenotype in Pancreatic Cancer
Source: Sci Rep. 2015 Jun 12;5:10867. doi: 10.1038/srep10867 (PMC4464260; doi:10.1038/srep10867)
Supplement: Supplementary Information [file srep10867-s1.doc]

**Caveolin-1 is Associated with Tumor Progression and Confers a Multi-Modality Resistance Phenotype in Pancreatic Cancer**

Moumita Chatterjee, Edgar Ben-Josef, Dafydd G. Thomas, Meredith A. Morgan, Mark M. Zalupski, Gazala Khan, Charles Andrew Robinson, Kent A. Griffith, Ching-Shih Chen, Thomas Ludwig, Tanios Bekaii-Saab, Arnab Chakravarti, Terence M. Williams

**Supplementary Data**

**Supplementary Figure S1. Presence of KRAS activating mutations increases Cav-1 expression.** Using two isogenic cell line pairs (SW48 and DLD-1), levels of Cav-1 and and GAPDH were assayed from asynchronously growing cells. The presence of a KRAS G13D mutant allele (“MUT”), results in increased basal expression levels of Cav-1. This experiment was performed multiple times with similar results.


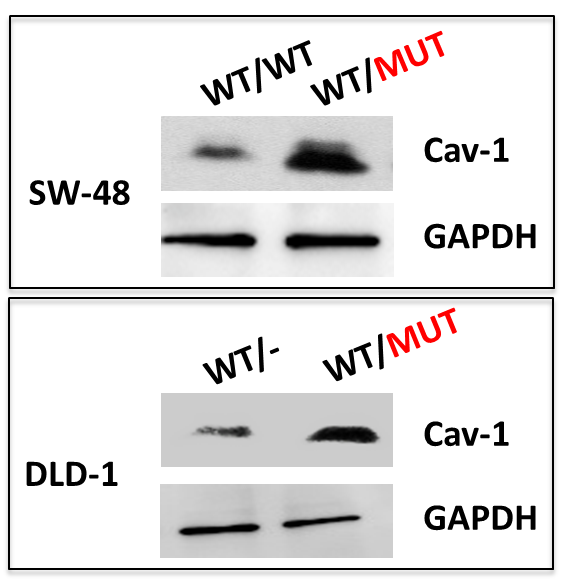


**Supplementary Figure 2. Loss of Cav-1 sensitizes cells to 5-FU.** WST-1 assays showing cell proliferation with percentage absorbance of siCav-1 treated cells normalized to scrambled control siRNA with a range of 5-FU concentrations in MIAPaCa-2 cells. Similar results were observed with BxPC3 cells also with 5-FU in the nanomolar range (*p<0.05).

**
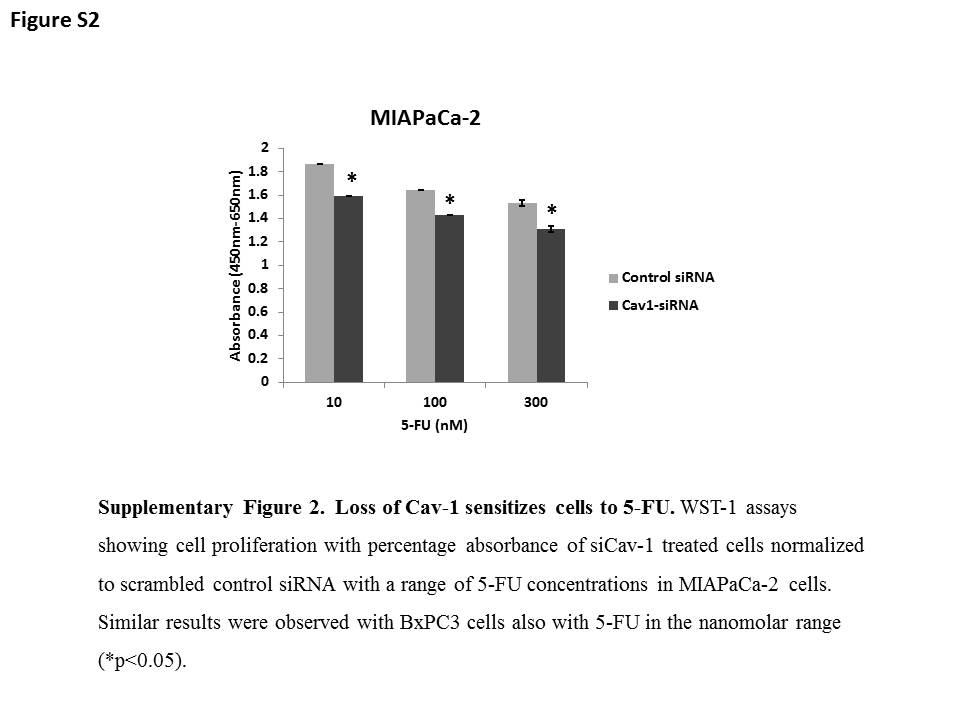
**

**Supplementary Figure S3: Caveolin-1 downregulation radiosensitizes cells.** Downregulation of Cav-1 with siRNA for 48 hours followed by radiation results in radiosensitization of MIAPaCa-2 and BxPC3 cells as measured by clonogenic assay. This experiment was performed multiple times with similar results. * p<0.05


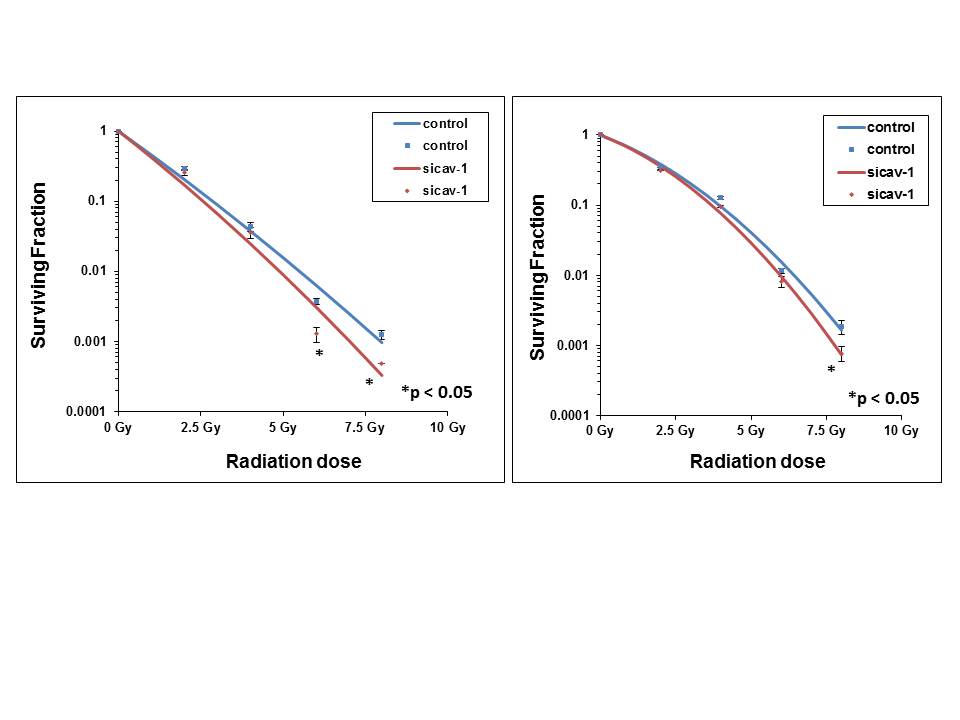


**MIAPaCa-2**

**BxPC3**

**Supplementary Table 1: Cell cycle analysis by DNA content on non-irradiated and irradiated control and stable Cav-1 knockdown (shCav-1) MIAPaCa-2 cell lines 24 hours after radiation (3Gy).** This experiment was performed multiple times with similar results.

| **Sample** | **G0/G1 (%)** | **S (%)** | **G2/M (%)** |
| --- | --- | --- | --- |
| **Control - 3Gy** | **84.7** | **7.9** | **6.7** |
| **Control + 3Gy** | **70.5** | **11.6** | **15.2** |
| **shCav-1 - 3Gy** | **83.4** | **7.4** | **8.1** |
| **shCav-1 + 3Gy** | **69.3** | **12.1** | **16.7** |
